# Correction: Genome-wide mutational landscape of mucinous carcinomatosis peritonei of appendiceal origin

**DOI:** 10.1186/s13073-014-0053-y

**Published:** 2014-07-26

**Authors:** Hakan Alakus, Michele L Babicky, Pradipta Ghosh, Shawn Yost, Kristen Jepsen, Yang Dai, Angelo Arias, Michael L Samuels, Evangeline S Mose, Richard B Schwab, Michael R Peterson, Andrew M Lowy, Kelly A Frazer, Olivier Harismendy

**Affiliations:** Division of Genome Information Sciences, Department of Pediatrics and Rady Children’s Hospital, University of California San Diego, La Jolla, CA USA; Division of Surgical Oncology, Department of Surgery, University of California San Diego, La Jolla, CA USA; Department of Medicine, University of California San Diego, La Jolla, CA USA; Bioinformatics Graduate Program, University of California San Diego, La Jolla, CA USA; Moores UCSD Cancer Center, University of California San Diego, La Jolla, CA USA; Department of Pathology, University of California San Diego, La Jolla, CA USA; Clinical and Translational Science Institute, University of California San Diego, La Jolla, CA USA; Institute for Genomic Medicine, University of California San Diego, La Jolla, CA USA; RainDance Technologies, Lexington, MA USA; Department of General, Visceral and Cancer Surgery, University of Cologne, Köln, Germany

## Correction

In the originally published version of this article [1], incorrect information about the corresponding authors was given. The following people should have been listed as corresponding authors: Andrew M Lowy, Kelly A Frazer, Olivier Harismendy. The corrected author information is shown above.
